# Hernie paraduodénale gauche: une cause rare d'occlusion intestinale

**DOI:** 10.11604/pamj.2017.28.32.13497

**Published:** 2017-09-14

**Authors:** Mohammed Alila, Abdelouahab Marouni, Imane Toughrai

**Affiliations:** 1 Service de Chirurgie Viscérale, Centre Hospitalier Universitaire Hassan II de Fès, Maroc

**Keywords:** Hernie interne, hernie paraduodénale, occlusion intestinale, Internal hernia, paraduodenal hernia, bowel obstruction

## Abstract

Nous rapportons l'observation de deux cas présentant une occlusion intestinale aiguë. L'intervention en urgence a trouvé une hernie para-duodénale gauche obstructive à l'origine d'un volvulus, avec nécrose des anses grêles incarcérées pour un patient, traité par résection anastomose en un temps et un grêle souffrant mais viable pour le deuxième malade, traité par la réduction du grêle incarcéré et l'obturation de l'orifice herniaire. Nous discutons à l'occasion de ces observations les particularités diagnostiques et thérapeutiques de cette affection rare.

## Abstract (en)

We here report two cases of acute bowel obstruction. Emergency intervention showed left obstructive paraduodenal hernia causing volvulus with necrosis of the incarcerated intestinal loops in one patient who underwent one-time anastomosis resection and suffering but viable small bowel in the second patient treated by incarcerated intestinal loop reduction and obturation of the orifice of the hernial canal. We here highlight the diagnostic and therapeutic features of this rare condition.

## Introduction

Les hernies internes sont rares [1]. Leur diagnostic est le plus souvent réalisé en peropératoire [2]. Les formes anatomiques de hernie interne sont nombreuses, certaines étant très rarement rapportées. Cependant, la connaissance des différentes variétés de hernies internes est fondamentale pour envisager un diagnostic préopératoire. La hernie para duodénale gauche de l'adulte est une forme rare de hernie interne [3, 4]. Nous présentons deux cas d'occlusion intestinale aiguë par hernie interne para duodénale gauche traités dans le service de chirurgie viscérale de CHU HASSAN II de FES, Maroc, afin de contribuer à la connaissance des particularités cliniques de cette entité.

## Patient et observation

**Figure 1:**
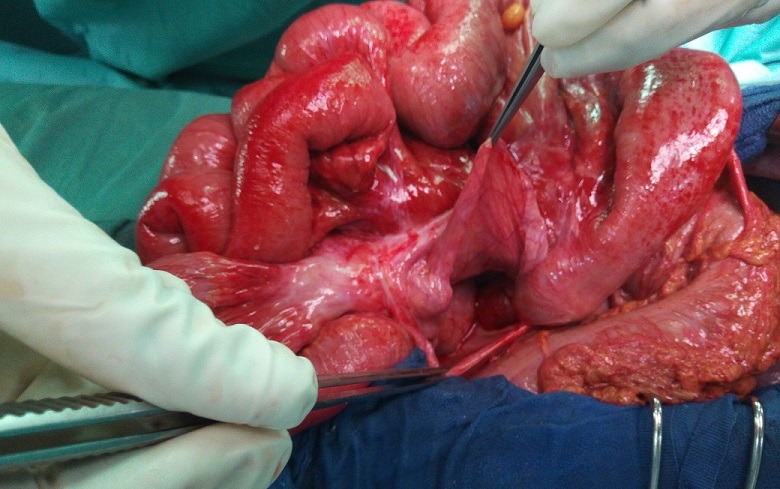
Vue opératoire de l'orifice d'une hernie para duodénales gauche

**Figure 2:**
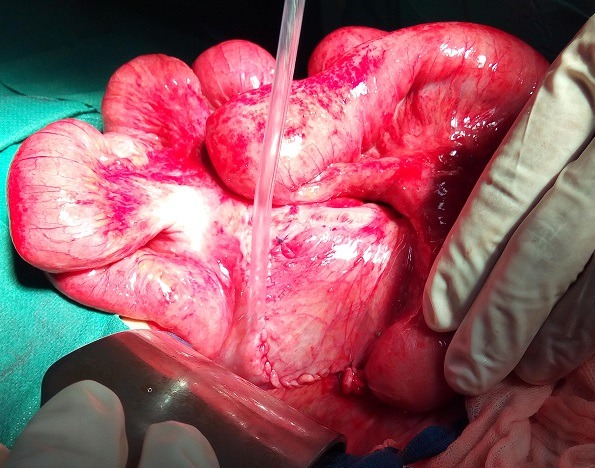
L'orifice d'une hernie para duodénales gauche après sa fermeture

**Observation 1**: Un homme de 50 ans a été admis en urgence; pour douleurs abdominales diffuses, vomissements alimentaires et arrêt des matières et des gaz. Cette symptomatologie évoluait depuis 48 heures. L'interrogatoire note la survenue régulière de crises similaires ayant cédé au bout de quelques heures. Il n'a pas été retrouvé d'antécédent de chirurgie abdominale, ni de traumatisme abdominal. L'examen physique a confirmé la présence d'un syndrome occlusif avec distension abdominale et météorisme. Les orifices herniaires pariétaux étaient libres. Le reste de l'examen physique était normal. La radiographie de l'abdomen sans préparation a noté des niveaux hydro-aériques de type grêlique. La tomodensitométrie n'a pas était réaliser suite a une insuffisance rénale fonctionnelle. Le diagnostic d'occlusion intestinale aiguë a été retenu. Mais devant l'aggravation de la sensibilité abdominale en défense; une laparotomie a été indiquée en urgence. L'incision a été une médiane. En per opératoire, nous avons noté une incarcération d'anses iléales à travers un défect d'environ 5 centimètres de long, situé au niveau de la fossette para duodénal gauche (Figure 1). L'iléon incarcéré était souffrant mais viable. Il s'agissait d'une occlusion intestinale aiguë par hernie interne para duodénal gauche. Le traitement a consisté à une réduction complète de son contenu par simple traction et une fermeture de l'orifice herniaire par un surjet de fil résorbable (Figure 2). Les suites opératoires ont été simples. La sortie de l'hôpital a été autorisée au troisième jour post opératoire.

**Figure 3:**
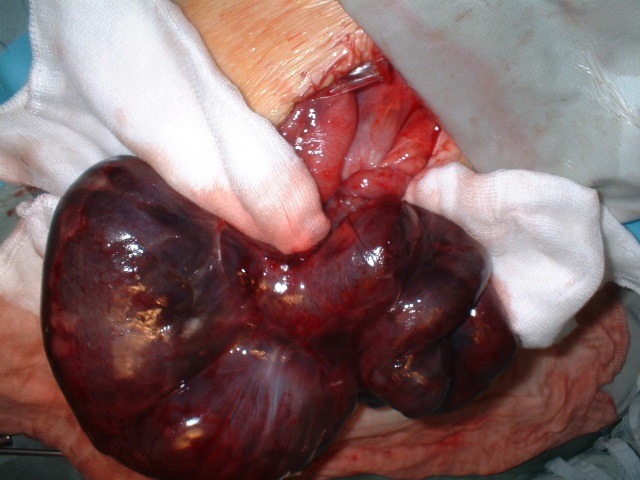
Hernie para duodénale gauche avec nécrose iléale

**Observation 2**: Une patiente de 18 ans, a été admise en urgence pour douleurs abdominales diffuses de survenue brutale. Aucun antécédent de chirurgie abdominale ou de traumatisme abdominal n'a été retrouvé à l'interrogatoire. L'examen physique a retrouvé une défense abdominale diffuse. Le diagnostic de péritonite aiguë généralisée a été évoqué. La radiographie de l'abdomen sans préparation a révélé la présence de niveaux hydro-aériques de type grêlique. L'hémogramme était normal en dehors d'un décompte leucocytaire à 18 800 éléments par millimètre cube. Le diagnostic préopératoire évoqué était une péritonite aiguë généralisée. Une laparotomie a été donc indiquée en urgence. Une anesthésie générale avec intubation orotrachéale a été conduite et la voie d'abord a été une médiane. A l'ouverture, la cavité péritonéale était le siège d'un épanchement sanguinolent. L'exploration a noté l'incarcération d'un segment de l'iléon dans un défect para duodénale, longé à droite par la veine mésentérique inférieure, L'iléon incarcéré était nécrosé sur 170 centimètres environ, situe à 2m50 de l'angle de treitz (Figure 3). Le traitement a consisté à une résection de l'iléon nécrosé, avec anastomose dans le même temps. Les suites opératoires ont été simples. L'alimentation liquide a été autorisée au quatrième jour et la sortie de l'hôpital a été au septième jour post opératoire.

## Discussion

Les hernies para-duodénales: sont les plus fréquentes des hernies internes, elles intéressent trois fois plus souvent l'homme que la femme [2, 3]. Les fossettes duodénales sont des replis péritonéaux qui peuvent relever de trois mécanismes, défaut d'accolement du péritoine, replis d'origine vasculaire (les vaisseaux soulèvent les feuillets péritonéaux créant ainsi les fossettes), ou bien encore ces deux mécanisme associés. Il existe alors cinq fossettes duodénales pouvant avoir un intérêt chirurgical [4]. Les hernies para duodénales gauches sont définies par une protrusion d'un viscère intra abdominal à travers la fossette paraduodénale décrite par Landzert [1, 4]. Devant une occlusion du sujet jeune sans antécédent de chirurgie ou de traumatisme abdominal, le diagnostic de hernie interne peut être évoqué, surtout lorsque l'interrogatoire retrouve un long passé de douleurs abdominales récurrentes. Leur diagnostic est généralement fait en per opératoire [2, 5]. Cependant, avec le développement de l'imagerie médicale et en particulier du scanner et de l'imagerie par résonnance magnétique, le diagnostic préopératoire est de nos jours possible [6]. En per opératoire, Le diagnostic de la hernie para duodénale gauche, peut être difficile, nécessite de repérer d'abord l'orifice herniaire, le collet est situé entre l'angle duodéno-jéjunal en haut et l'artère mésentérique inférieure en bas, alors que le bord libre du collet contient la veine mésentérique inférieure et son identification participe également au diagnostic [1, 2]; le sac alors est rétro-mésocolique. En effet la majorité de ces hernies sont à collet large et peu serré, permettant ainsi d'obtenir une réduction complète de son contenu par simple traction et l'orifice herniaire doit être fermé à l'aide de fils résorbables on non. Mais toute tentative d'excision du sac herniaire doit être proscrire [2, 7].

## Conclusion

La hernie para duodénale gauche est une cause rare, mais possible d'occlusion intestinale aiguë chez l'adulte. Il convient d'y penser devant la présence d'épisodes de sub-occlusion spontanément réduites. Le diagnostic tardif peut occasionner des complications à type de nécrose d'anse.

## Conflits d’intérêts

Les auteurs ne déclarent aucun conflit d'intérêts.
